# Effect of Ultrasound on the Green Selective Oxidation of Benzyl Alcohol to Benzaldehyde

**DOI:** 10.3390/molecules24224157

**Published:** 2019-11-16

**Authors:** Marion L. Chevallier, Sarah Dessolin, Fanny Serres, Lucile Bruyas, Gregory Chatel

**Affiliations:** Univ. Savoie Mont Blanc, LCME, F-73000 Chambéry, France; marion.chevallier@univ-smb.fr (M.L.C.); sarah.dessolin@orange.fr (S.D.); serresfanny@orange.fr (F.S.); lucile.bruyas@gmail.com (L.B.)

**Keywords:** sonochemistry, ultrasound, hydroxyl radical, selective oxidation, benzyl alcohol, benzaldehyde

## Abstract

Oxidation of alcohols plays an important role in industrial chemistry. Novel green techniques, such as sonochemistry, could be economically interesting by improving industrial synthesis yield. In this paper, we studied the selective oxidation of benzyl alcohol as a model of aromatic alcohol compound under various experimental parameters such as substrate concentration, oxidant nature and concentration, catalyst nature and concentration, temperature, pH, reaction duration, and ultrasound frequency. The influence of each parameter was studied with and without ultrasound to identify the individual sonochemical effect on the transformation. Our main finding was an increase in the yield and selectivity for benzaldehyde under ultrasonic conditions. Hydrogen peroxide and iron sulfate were used as green oxidant and catalyst. Coupled with ultrasound, these conditions increased the benzaldehyde yield by +45% compared to silent conditions. Investigation concerning the transformation mechanism revealed the involvement of radical species.

## 1. Introduction

In industrial chemistry, selective oxidation is commonplace as it enables the synthesis of carbonyl compounds, alcohols, epoxides, carboxylic acids, ethers, and esters [1] that are used in various fields, such as agroindustry, pharmaceuticals, cosmetics, and dyes. However, some processes developed in laboratories are not suitable for industrial applications due to safety concerns, like thermal hazards and hazardous waste production [1,2,3]. For these reasons, sustainable activation techniques that would enable the use of abundant, cheap, and safe reagents must be developed.

Sonochemistry is the use of power ultrasound (US) as an activation method to perform chemical reactions [4]. It can induce changes of reactivity, enhance transformations by improving yields or selectivity, reduce duration time, and eventually replace hazardous reagents with harmless ones [5,6,7,8,9]. Ultrasonic waves propagate in liquids, which provokes compression and dilatation of the medium. When the dilated medium reaches a pressure below the saturated vapor pressure, a microbubble of gas is created, and the following cycles of compression and decompression lead to its growth until it reaches a critical size and implodes. The implosion of the bubble, known as cavitation phenomenon, causes extreme temperature (1000–5000 K) and pressure (1000 atm) [10]. Physical and chemical effects can be observed during a cavitation phenomenon. Notable physical effects include microjets stream shockwaves and improved mass transfer, while chemical effects arise from radical species formation due to homolytic breakage of molecules [11]. In the case of water as a solvent, hydroxyl radical species (HO^•^) are generated [12]. These species are powerful oxidants (E° = 2.3 V) and are highly reactive towards organic compounds (k = 10^6^ − 10^10^ mol L^−1^.s^−1^) [13].

Benzaldehyde is an important organic intermediate in pharmaceutical, fragrances, dyes, and agribusiness industries. In the pursuit of more sustainable chemistry, the 12 principles of green chemistry were proposed [14] and suggest using safer solvents and auxiliaries in chemical synthesis. In organic chemistry, the use of harmful and polluting oxidants [15] should be replaced by harmless, environmentally safe, and abundant oxidants. Thus, several environmentally driven studies focused on benzaldehyde synthesis using H_2_O_2_ as a green oxidant [16,17,18,19]. Sonochemistry has proven a promising technique to reduce chemicals amounts, limit reaction duration, avoid extreme conditions, and lead to energy saving [11,20,21].

In this study, we searched for simple conditions to convert benzyl alcohol to benzaldehyde using green oxidants: Molecular dioxygen and hydrogen peroxide [2]. To this end, various parameters such as substrate concentration, oxidant concentration, catalyst nature and concentration, temperature, pH, and reaction duration were studied over wide ranges to identify optimal conditions for the transformation. We tested molecular dioxygen and hydrogen peroxide as oxidants because of their clean nature [22,23]. The transformation mechanism was investigated under low and high frequency ultrasound.

## 2. Results and Discussion

In the following section, the oxidation of benzyl alcohol into benzaldehyde is described using three parameters: Benzyl alcohol conversion, benzaldehyde yield, and selectivity for benzaldehyde. These are calculated according to the following equations (Equations (1)–(3))

(1) $$Conversion\;\left(\%\right)=\frac{moles\;of\;benzyl\;alcohol\;reacted}{moles\;of\;benzyl\;alcohol\;introduced}$$

(2) $$Benzaldehyde\;yield\;\left(\%\right)=\frac{moles\;of\;benzaldehyde\;produced}{moles\;of\;benzyl\;alcohol\;introduced}$$

(3) $$Selectivirty\;for\;benzaldehyde\;\left(\%\right)=\frac{moles\;of\;benzaldehyde\;produced}{moles\;of\;benzyl\;alcohol\;reacted}$$

### 2.1. Effect of Substrate Concentration

The effect of benzyl alcohol concentration in water (6.37 mL batch) on its conversion rate and benzaldehyde yield was evaluated in the presence of 1 and 0.01 molar equivalent of the oxidant (aqueous 30% H_2_O_2_ solution) and the catalyst (FeSO_4_), respectively. The following substrate concentrations were tested: 286 mM, 571 mM, 1.14 M, and 2.26 M, and the results are presented in Table 1. The transformations in silent conditions (without ultrasound under argon bubbling stirring) and under low-frequency US (20 kHz) showed similar tendencies. Benzyl alcohol conversion rates reached the highest range (from 44% to 59%) at a substrate concentration of 571 mM. In these conditions, the conversion of benzyl alcohol is only slightly affected by initial substrate concentration, but when it is increased from 571 mM to 2.26 M, benzaldehyde yield drops from 13% (571 mM) to 7% (2.26 M) under silent conditions, and from 21% (571 mM) to 7% (2.26 M) under ultrasonic conditions.

Under low-frequency US irradiation (20 kHz), the benzyl alcohol conversion rate and benzaldehyde yield are higher than in silent conditions, especially at lower concentrations (about +8% conversion and +40% yield at 571 mM of benzyl alcohol) compared to the experiment run at a concentration of 2.26 M, for which the conversion rates and yields are similar in both silent and ultrasonic conditions. When the power of US activation is the same under all conditions, the effect of US has a greater impact on a lower amount of substrate dissolved in a fixed volume of reaction medium.

It appears that 571 mM is the optimal concentration to convert benzyl alcohol to benzaldehyde. Further parameter studies were performed at this concentration.

### 2.2. Effect of Oxidant in the Reaction Medium

Clean oxidants such as molecular dioxygen (O_2_) and hydrogen peroxide (H_2_O_2_) were tested. Although molecular dioxygen has been successfully employed in aerobic oxidation of alcohols [24], dioxygen bubbling in the presence of FeSO_4_ as the catalyst (1% molar) did not lead to benzyl alcohol conversion (571 mM of benzyl alcohol; 120 min; 70 °C; US conditions: 20 kHz, P_acous_ = 47.9 W L^−1^ h^−1^, 13 mm probe). In the same conditions, hydrogen peroxide did lead to benzyl alcohol transformation. The influence of its concentration and its introduction mode on conversion rate and yield were further studied.

#### 2.2.1. Effect of H_2_O_2_ Concentration

**Figure 1:**
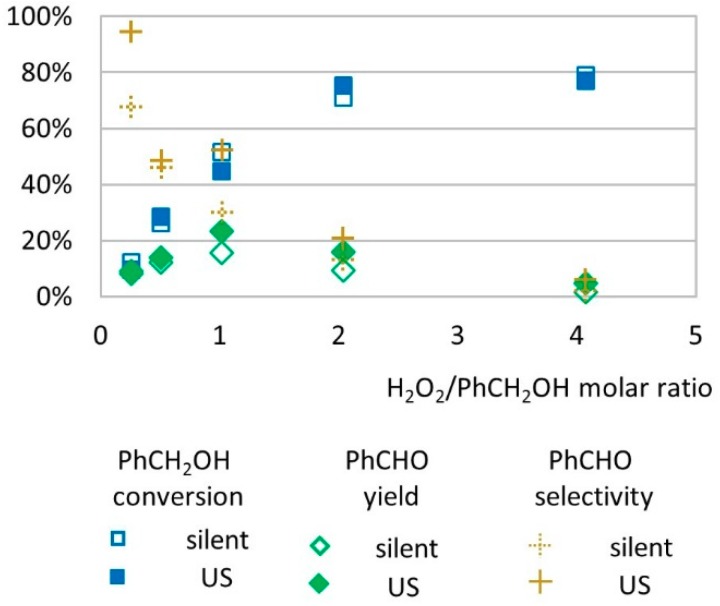
Oxidation of PhCH_2_OH as a function of H_2_O_2_/substrate molar ratio in silent conditions and under US irradiation (20 kHz). Reaction conditions: 571 mM PhCH_2_OH, 1% molar eq. FeSO_4,_ 5.61 mL H_2_O, 62 °C, 15 min. Silent conditions: Argon bubbling; US conditions: 20 kHz, P_acous_ = 47.9 W L^−1^ h^−1^, 13 mm probe.

The effect of H_2_O_2_ molar ratio on the conversion rate and benzaldehyde yield was studied on a range of 0.25 to 4 molar equivalents related to the benzyl alcohol substrate (Figure 1).

In this experiment, reactions operated in silent conditions and under low-frequency US (20 kHz) showed similar trends. The benzyl alcohol conversion rate increased as higher concentrations of H_2_O_2_ was introduced in the medium (from 12% at 0.25 molar equivalent of H_2_O_2_ to 79% at 4 molar equivalents of H_2_O_2_ under silent conditions and from 10% at 0.25 molar equivalent of H_2_O_2_ to 77% at 4 molar equivalents of H_2_O_2_ under US). Benzaldehyde yield increased from 9% to 23% when the H_2_O_2_/substrate molar ratio increased from 0.25 to 1 under US and decreased to 5% at 4 molar equivalents of H_2_O_2_ (Figure 1). Below 1 molar equivalent of H_2_O_2_, the limited concentration of oxidizing species in the medium seems to promote good selectivity to benzaldehyde, by limiting the over-oxidation phenomena that can occur at higher H_2_O_2_ concentration [25,26]. In that case, hydroxyl radical species formed by homolytic cleavage of hydrogen peroxide through the Fenton process are the limiting agent to benzyl alcohol oxidation. For experiments performed with 1 to 4 molar equivalent of H_2_O_2_, the conversion of alcohol increased by +71% under US irradiation and by +52% in silent conditions while the yield in benzaldehyde was reduced by −78% both in US and silent conditions. This decrease in benzaldehyde yield is presumably due to further oxidation of benzaldehyde into other products. Among other products, benzoic acid was identified at concentration levels corresponding to less than 1% yield. The identification of other transformation products is further discussed in this study.

#### 2.2.2. Effect of H_2_O_2_ Introduction Mode in the Reaction Medium

**Figure 2:**
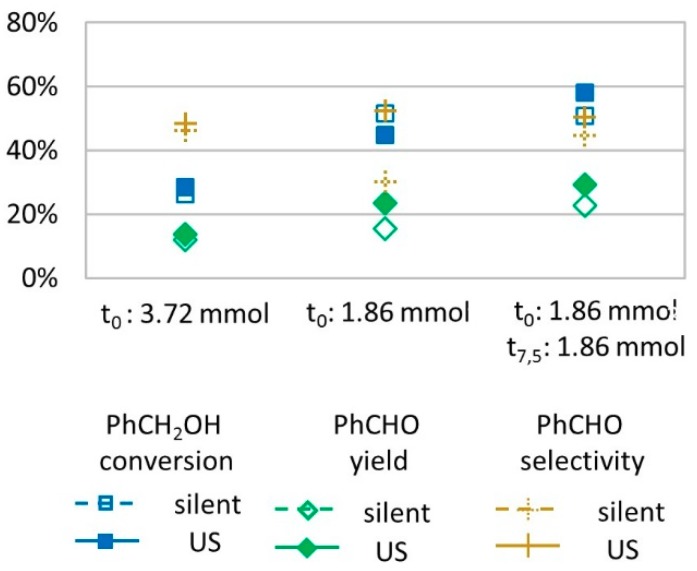
Oxidation of PhCH_2_OH as a function of the H_2_O_2_ introduction mode in silent condition and under US irradiation (20 kHz). Reaction conditions: 571 mM PhCH_2_OH, 1 molar eq. H_2_O_2_; 1% molar eq. FeSO_4,_ 5.61 mL H_2_O, 70 °C, 15 min); silent conditions: Argon bubbling; US conditions: 20 kHz, P_acous_ = 47.9 W L^−1^ h^−1^, 13 mm probe.

Considering that H_2_O_2_ may decompose during the reaction with the targeted substrate, we performed experiments in which the oxidant was not introduced all at once. Instead, the molar amount of H_2_O_2_ (3.72 mmol) was divided into two portions, of 1.86 mmol each, that were introduced at *t* = 0 min and at *t* = 7.5 min. For both introduction modes (all at once and portioned), similar conversions and yields were observed (Figure 2), suggesting that H_2_O_2_ introduction mode is not the limiting factor to further conversion of benzyl alcohol into benzaldehyde.

### 2.3. Effect of the Catalyst Nature and Concentration

#### 2.3.1. Effect of the Catalyst Nature

Catalyst complexes implying transition metals are often considered as efficient catalysts especially for oxidation reactions [27,28,29,30,31,32,33]. The effect of several metal oxides currently used as catalysts (Fe_3_O_4_, FeO, Fe_2_O_3_, FeTiO_3_, CoFe_2_O_4_, MnTiO_3_), metals (Cu(0) and Fe(0)), and salts (MnCl_2_, CuCl_2_, CuSO_4_, FeCl_3_, and FeSO_4_) were tested in the transformation of benzyl alcohol to benzaldehyde (Table 2) in the above conditions. When the 15 min reaction was conducted with metal oxides as the catalyst, an average of 10% conversion of benzyl alcohol was observed but only traces of benzaldehyde were obtained. Transformations with salts containing Mn and Cu as transition metals led to higher benzaldehyde yields (3–8%) with the same benzyl alcohol conversion rates (close to 10%). Salts containing Fe (FeSO_4_ and FeCl_3_) lead to higher conversion rates (40% and 53%, respectively) and higher yields (26% and 17%, respectively). Dissociated salts appeared to be the most efficient form of catalyst, for oxidation in these aqueous conditions with H_2_O_2_ as the oxidizing agent, leading to better conversions and yields. For example, FeSO_4_ dissociates into Fe^2+^ and SO_4_^2−^; iron(II) ions are known to react with H_2_O_2_ to produce hydroxyl radical species according to the Fenton process (Equations (4)–(6)), [34] and regenerate forming HO_2_^●^ radical species and protons from hydrogen peroxide.

Fe^2+^ + H_2_O_2_ → Fe^3+^ + HO^-^ + HO (4)

Fe^3+^ + H_2_O_2_ → Fe^2+^ + HO_2_^●^ + H^+^ (5)

Fe^3+^ + HO_2_^●^ → Fe^2+^ + O_2_ + H^+^ (6)

In Table 2, one can observe that iron salts, through this Fenton effect, are more effective in the transformation of benzyl alcohol than manganese and copper salts. A study that tested CuSO_4_ reported an almost complete transformation of benzyl alcohol (99%) with 76% benzaldehyde yield in an aqueous medium while using 3 molar equivalents of H_2_O_2_ [17]. In this case, mechanistic investigation indicated that no hydroxyl radical species were produced.

A complementary study allowed us to compare benzyl alcohol transformation into benzaldehyde in the presence of different salts (FeSO_4_, FeCl_3_, CuSO_4_, CuCl_2_, and MnCl_2_) as well as iron and copper solid powders with 0.5, 1, and 2 molar equivalents of hydrogen peroxide to substrate (Table 3). These experiments showed no global tendency in the transformations of benzyl alcohol with various salts as a function of the H_2_O_2_ amount. In fact, the optimal amount of H_2_O_2_ is specific to each salt. The use of FeSO_4_ as catalyst under low-frequency US (20 kHz) led to the highest benzaldehyde yield (30%) with an interesting selectivity of 60% at 1 molar equivalent of H_2_O_2_. In most cases, activation by US slightly decreased benzyl alcohol conversion (by about −6%) and increased benzaldehyde yields (by about +74%) compared to silent conditions. After 15 min under US activation, an emulsion was observed contrary to the reaction medium without US, most likely provoked by the physical effects of low-frequency US irradiation [35]. Experiments conducted with Cu(0) as catalyst under US led to 17% conversion, which is similar to experiments conducted with Cu salts (CuSO_4_ and CuCl_2_: 11% and 16%, respectively); whereas benzaldehyde yield with Cu(0) was two times lower compared to experiments conducted with CuSO_4_ and CuCl_2_ salts as catalysts (4%, 8%, and 7% yield, respectively). For zero valent iron powder, up to 3 molar equivalents of H_2_O_2_ were added, resulting in low conversion (11%) and low benzaldehyde formation (2% yield). The transformation did not lead to the same high conversion rates (43% and 53%) and yields (26% and 17%) obtained with FeSO_4_ and FeCl_3_ salts.

#### 2.3.2. Effect of the Catalyst Amount

**Figure 3:**
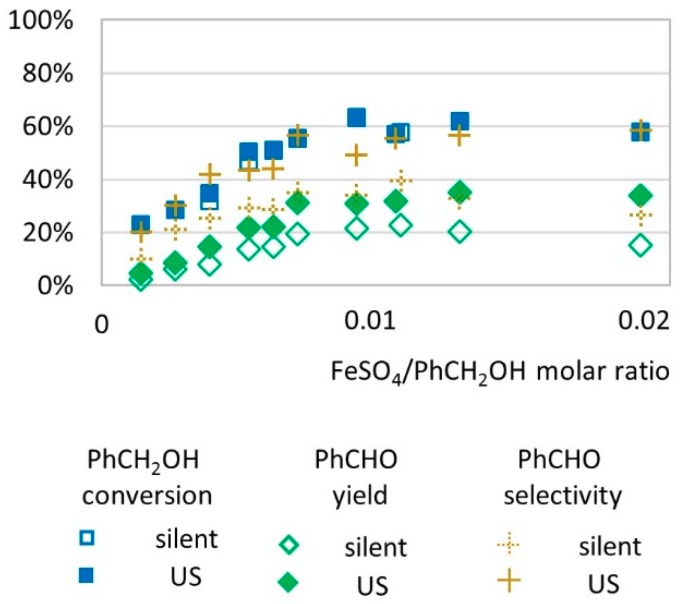
Oxidation of PhCH_2_OH as a function of molar ratio catalyst/substrate in silent condition and under US irradiation (20 kHz). Reaction conditions: 571 mM PhCH_2_OH, 1 molar eq. H_2_O_2_; 5.61 mL H_2_O, 70 °C, 15 min. Silent conditions: Argon bubbling; US conditions: 20 kHz, P_acous_ = 47.9 W L^−1^ h^−1^, 13 mm probe.

We investigated the influence of catalyst amount (from 0.001 to 0.02 molar equivalent relative to substrate) on the transformation yield of benzyl alcohol to benzaldehyde (Figure 3). Increasing the catalyst amount (from 0.001 to 0.01 molar ratio) led to higher conversion and higher yields with or without US activation. From 0.01 to 0.02 molar ratio, in silent conditions, benzaldehyde yield decreased as catalyst concentration increased, whereas under US irradiation, benzaldehyde yield was not affected by the amount of catalyst. In these conditions, activation by low-frequency US increases the selectivity of the transformation to benzaldehyde (58% under US vs. 26% under silent conditions with 0.02 FeSO_4_/PhCH_2_OH molar ratio).

### 2.4. Effect of pH

The effect of pH was studied for the transformation of benzyl alcohol to benzaldehyde. The pH of the reaction medium was measured, before and after the catalyst introduction. An acidification of the medium from 6.5 to 5.2 occurred when FeSO_4_ was added to water and benzyl alcohol. A further decrease in pH value from 5.2 to 2.7 was noted when H_2_O_2_ was introduced. During the reaction, pH remained at 2.7.

**Figure 4:**
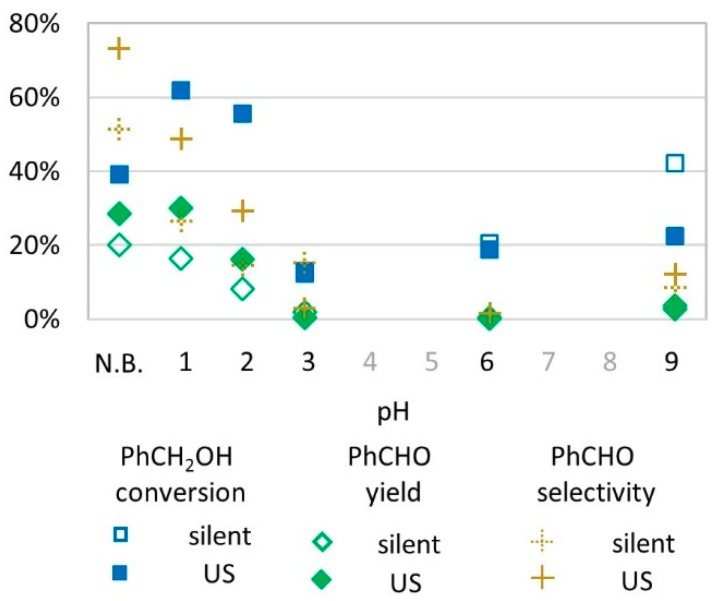
Oxidation of PhCH_2_OH as a function of pH (buffered or not) in silent condition and under US irradiation (20 kHz). Reaction conditions: 571 mM PhCH_2_OH, 1 molar eq. H_2_O_2_; 1% molar eq. FeSO_4_, 5.61 mL H_2_O, 70 °C, 15 min. In non-buffered (N.B.) conditions and at pH 1, 2, and 3, empty blue square and full blue square overlap.

To test the influence of pH in the conversion of benzyl alcohol to benzaldehyde, aqueous acidic and basic buffers were prepared at pH = 1, 2, 3, 6, and 9 and replaced water in the transformation (Figure 4). It appears that acidic media (pH 1–2) led to high conversion (60%) and yields (up to 30% at pH 1 under US activation) whereas at pH = 3, conversion was lower (10%) and did not lead to benzaldehyde. At pH = 6, the benzyl alcohol conversion rate reached 20% but with no formation of the desired benzaldehyde for both US and silent conditions. At pH = 9, the conversion rate was higher in silent conditions (40% vs. 20% under US activation) while benzaldehyde yield was evaluated at 5% in both conditions.

**Figure 5:**
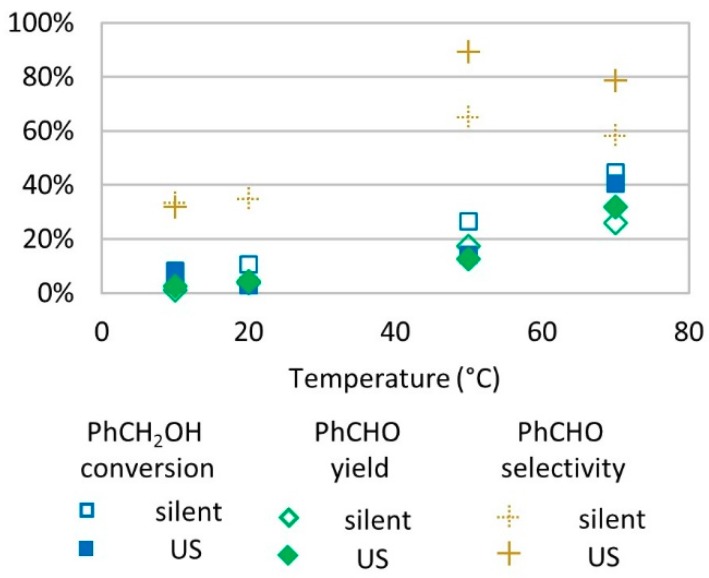
Oxidation of PhCH_2_OH as a function of temperature in silent condition and under US irradiation (20 kHz). Reaction conditions: 571 mM PhCH_2_OH, 1 molar eq. H_2_O_2_; 1% molar eq. FeSO_4_, 5.61 mL H_2_O, 15 min. Silent conditions: Argon bubbling; US conditions: 20 kHz, P_acous_ = 47.9 W L^−1^ h^−1^, 13 mm probe.

The effect of pH can be related to potential-pH diagrams as chemical predominant species are determined by the medium conditions [36]. For pH values above 4, ferrous ions (Fe(II)) are unstable and form ferric ions (Fe(III)) that tend to bind into mineral metal oxide structures and are thus not available as ions to participate in radical species formation [37]. Medium pH values also acts on H_2_O_2_ stability [38], since in basic media, H_2_O_2_ decomposes to water and oxygen (Equation (7), explaining very low yields obtained at pH 9 (Figure 5).

2 H_2_O_2_ + 2 HO^−^ ⇌ 2 HOO^−^ + 2 H_2_O → O_2_ + 2 HO^−^ + 2 H_2_O (7)

### 2.5. Effect of Temperature

The oxidation of benzyl alcohol was tested under different temperatures ranging from 10 to 70 °C. It was observed that benzyl alcohol conversion and benzaldehyde yield increased with temperature. Below 20 °C, only low conversion rates were observed (<10%). At 50 °C and 70 °C, US conditions led to higher selectivity for benzaldehyde in the oxidation of benzyl alcohol (Figure 5).

One explanation of better conversion and yields at a higher temperature is that the enhanced agitation and molecule movement increase the probability for radical species and benzyl alcohol to meet. The decrease in selectivity for benzaldehyde from 50 °C to 70 °C is caused by a higher increase in conversion (+68% in silent conditions and +188% under US irradiation) compared to yield (+51% in silent conditions and +155% under US irradiation). The higher increase in conversion than in yield may be due to benzyl alcohol conversion into other undetected products or the conversion of benzyl alcohol into benzaldehyde followed by a degradation of the product, leading to a lower yield. Several studies described that the transformation of benzyl alcohol into benzaldehyde under temperature ranging from 39 °C to reflux [16,17,18,19,32,33,39,40,41,42,43] and at a low temperature (15 °C) led to much slower formation of benzaldehyde [39]. Even though H_2_O_2_ degrades faster in higher temperatures, which eventually decreases oxidizing power of the medium, it is not the case in our tested conditions. Experiments on temperature effects were conducted at 1 molar equivalent relative to substrate (about 2% wt. concentration in water). In this condition, the adiabatic decomposition temperature is inferior to 89 °C [38], which explains why no negative effect was observed on benzyl alcohol conversion and benzaldehyde production in the studied range of temperatures of 10 to 70 °C.

### 2.6. Kinetics of Benzyl Alcohol Transformation: Effect of Reaction Time

**Figure 6:**
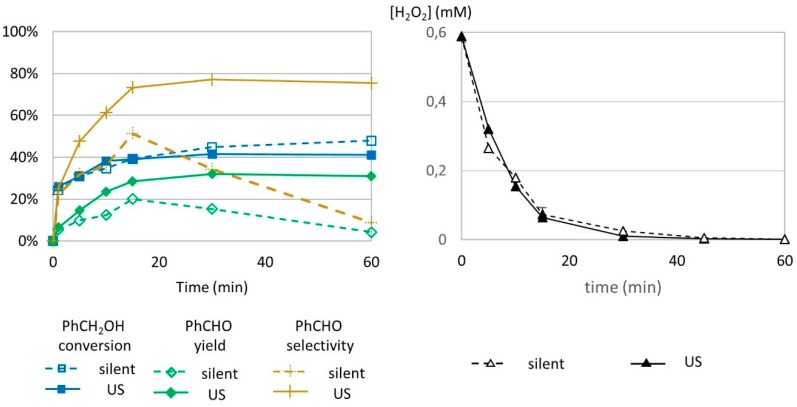
Oxidation of PhCH_2_OH as a function of time in silent condition and under US irradiation (20 kHz). Reaction conditions: 571 mM PhCH_2_OH, 1 molar eq. H_2_O_2_, 1% molar eq. FeSO_4_, 5.61 mL H_2_O, 70 °C (left) and monitoring of H_2_O_2_ over time. Reaction conditions: 584 mM H_2_O_2_; 5.5 mM FeSO_4_; 5.988 mL water (right). Silent conditions: Argon bubbling; US conditions: 547 kHz, P_acous_ = 55.5 W L^−1^ h^−1.^

To investigate the effect of reaction duration on benzyl alcohol transformation, it is important to consider the moderate solubility of the substrate, benzyl alcohol, and its main product, benzaldehyde, in water, respectively 397 mM (42.9 g L^−1^ in water at 25 °C) and 65.6 mM (6.95 g L^−1^ in water at 25 °C) [44]. Because the benzyl alcohol initial concentration is superior to the solubility threshold in water and benzaldehyde’s maximum solubility in water at 25 °C corresponds only to 11.5% yield, samplings of the aqueous medium would not allow for accurate representation of the transformation state. Therefore, the whole transformation medium was fully solubilized in acetonitrile (1:1 *v*/*v*) to ensure reliable analysis. Sacrificial monitoring was performed at 0, 5, 10, 15, 20, 30, and 60 min. Benzyl alcohol conversion increased with time from 0% to an average of 48% at 60 min under silent conditions and 41% under US irradiation (Figure 6, left). Results with and without US irradiation are similar, where only a slight increase in benzyl alcohol conversion is observable under US conditions. Benzaldehyde yield increased over the course of 15 min in both conditions with superior values under US (20% in silent conditions vs. 29% under US irradiation, which corresponds to a +45% increase in yield when the reaction medium is under US irradiation). After 15 min, different trends were observed for both silent and US conditions. (i) In silent conditions using a 6.366 mL reaction volume, benzaldehyde yield decreased and reached 4% at 60 min. This is due to further transformation of formed benzaldehyde in other compounds (which is discussed later in the study). (ii) Under US irradiation, benzaldehyde yield remained stable (29% at 15 min and 31% at 60 min).

The effect of low-frequency US is clearly observable in this study. In silent conditions, formed benzaldehyde could be transformed into other products, while under 20 Hz US irradiation, it was protected from degradation. Since the conversion rate for benzyl alcohol remained stable from 15 to 60 min, it excluded the possibility of simultaneous production and degradation. Chakma et al. studied the individual mechanisms of sonolysis and the Fenton process in the hybrid sono-Fenton process [35]. They concluded that only physical effects contributed to improvement in organic dye degradation and identified intense mixing as the cause of the positive synergy. Microagitation, turbulence, and other physical phenomena are critical for efficient mixing, since they lead to a better homogeneous repartition of hydroxyl radical species and higher probabilities for radical and organic species to meet, compared to the Fenton process for which radical distribution depends on agitation created by argon bubbling.

In order to evaluate the role of US on H_2_O_2_ decomposition rate, H_2_O_2_ concentration was monitored in the reaction medium without the substrate by indirect titration with MnO_4_^−^ according to the following equation:

2 MnO_4_^−^ + 6 H^+^ + 5 H_2_O_2_ → 2 Mn^2+^ + 8 H_2_O + 5 O_2._ (8)

Results of hydrogen peroxide titration are presented in Figure 6 (right). No difference in concentration of H_2_O_2_ could be observed under US irradiation or in silent conditions, indicating that low-frequency US does not impact H_2_O_2_ decomposition.

### 2.7. Effect of US Frequecy

**Figure 7:**
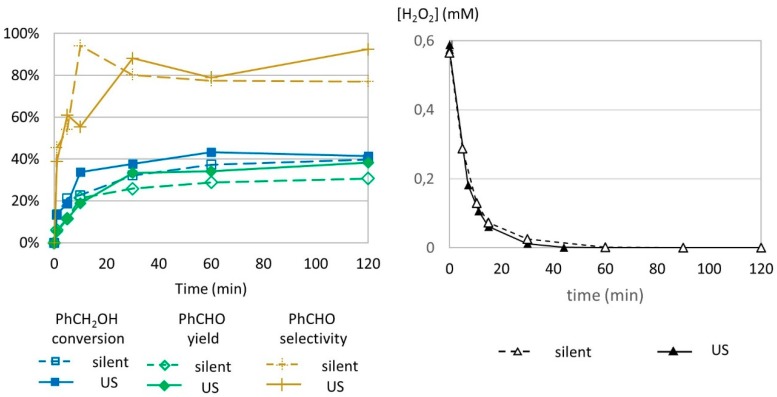
Oxidation of PhCH_2_OH as a function of time in silent condition and under US irradiation (547 kHz). Reaction conditions: 571 mM PhCH_2_OH, 1 molar eq. H_2_O_2_; 1% molar eq. FeSO_4_, 56.1 mL H_2_O, 70 °C (left) and monitoring of H_2_O_2_ over time. Reaction conditions: 584 mM H_2_O_2_; 5.5 mM FeSO_4_; 5.988 mL water (right). Silent conditions: Argon bubbling; US conditions: 547 kHz, P_acous_ = 55.5 W L^−1^ h^−1^.

Ultrasound frequency was tested as another parameter for benzyl alcohol transformation to benzaldehyde. High-frequency US (547 kHz) was provided by a cup horn device that required at least 60 mL volume to operate. For this reason, the reaction volume was adapted to 63.66 mL, which corresponded to a proportional increase by a factor of 10 for each reagent compared to low frequency operating conditions (6.366 mL). Benzyl alcohol (571 mM), H_2_O_2_ (1 molar equivalent), and FeSO_4_ (1% molar equivalent) were introduced in the exact same proportions as in previously described reactions. Sacrificial experiments were set up according to parameters explained above. The reaction was analyzed until 120 min instead of 60 min to reach stable values for conversion and yield (Figure 7).

The benzyl alcohol conversion profiles in silent conditions and under US irradiation at 547 kHz are very similar to the conversion profiles under 20 kHz US irradiation (Figure 7, left). In silent conditions, a higher reaction volume (63.66 mL) did not show degradation of the formed benzaldehyde after obtaining the maximum yield, contrary to the trends observed in a smaller volume (6.366 mL). This suggests that volume has an effect in benzaldehyde transformation.

The decomposition of H_2_O_2_ was studied by indirect titration as previously described (Figure 7, right). No difference in H_2_O_2_ concentration was observed, leading to the conclusion that high-frequency US does not impact the decomposition of hydrogen peroxide.

### 2.8. Study of the Transformation Mechanism

#### 2.8.1. Radical Species Involvement in the Transformation Mechanism

In this study, the effect of low-(20 kHz) and high-(547 kHz) frequency US irradiations were tested on the simple system composed of benzyl alcohol, the substrate to be converted; water as the solvent; H_2_O_2_ as the oxidant; and FeSO_4_ as the catalyst. Fenton reagents are known to generate hydroxyl radicals HO^•^ [45,46,47] that react quickly with other radicals or organic compounds such as benzyl alcohol and benzaldehyde (k = 8.4 × 10^9^ mol L^−1^ s^−1^ and 4.4 × 10^9^ mol L^−1^ s^−1^, respectively) [13]. On the other hand, US and, more specifically, high-frequency US have been described to generate radical species such as hydroxyl radicals in water [7]. The quantification of HO^•^ in our systems was estimated by KI dosimetry method. The reaction presented in Equation (8) was monitored by UV-vis spectrophotometry [48].

2 HO^•^ + 3 I^−^→ 2 HO^−^ + I_3_^−^ (9)

During irradiation at 547 kHz, we evaluated that 2.4 × 10^−4^ mol L^−1^ h^−1^ of HO^•^ were produced compared to 7.6 × 10^−8^ mol L^−1^ h^−1^ at 20 kHz (3200 times less). *Ter*t-butanol is a known HO^•^ scavenger according to Equation (10) [49,50], which we used in our system to assess the role of radical species in the transformation mechanism.

(CH_3_)_3_COH + OH^•^→ (CH_3_)_2_CH_2_^•^COH + H_2_O (10)

**Figure 8:**
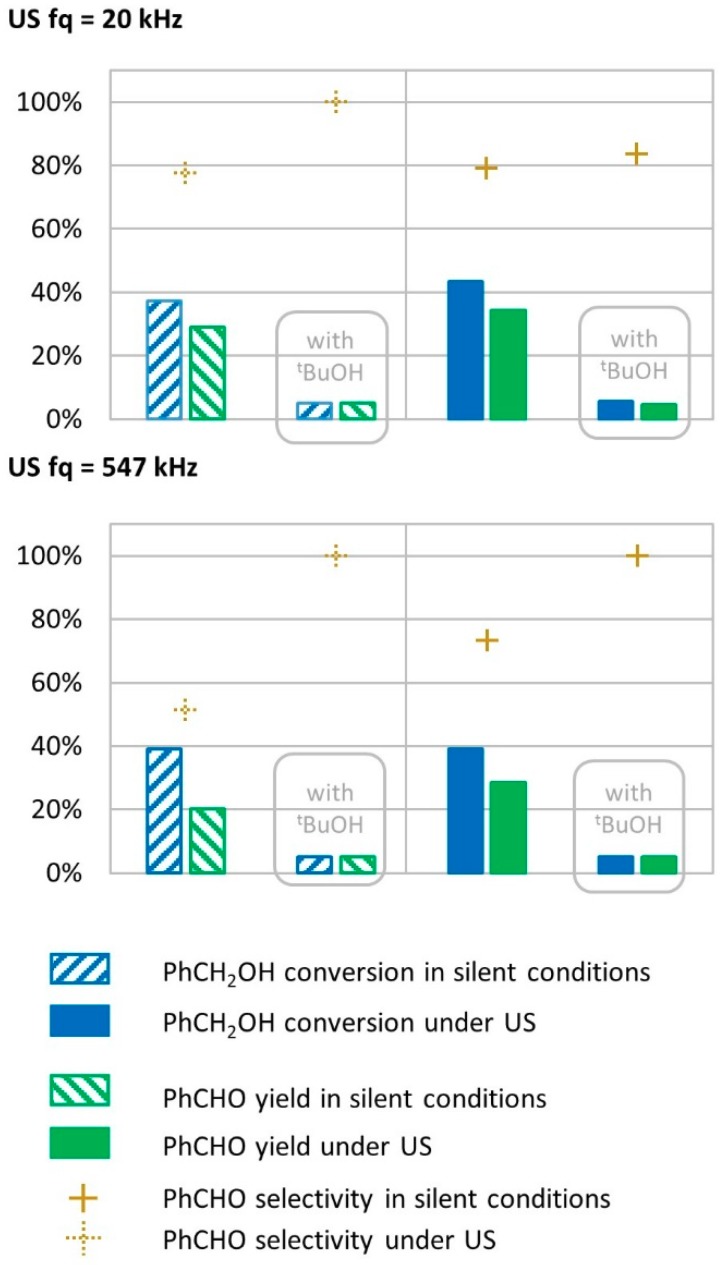
Oxidation of PhCH_2_OH with *tert-*butanol as radical inhibitor in silent condition and under US irradiation (20 kHz and 547 kHz). Reaction conditions under 20 kHz and silent conditions; 571 mM PhCH_2_OH, 1 molar equivalent of H_2_O_2_, 1% molar equivalent of catalyst, 5.61 mL H_2_O, 70 °C, 15 min. Silent conditions: Argon bubbling; US conditions: 20 kHz, P_acous_ = 47.9 W L^−1^ h^−1^, 13 mm probe; under 547 kHz and in silent conditions: 571 mM PhCH_2_OH, 1 molar eq. H_2_O_2_; 1% molar eq. FeSO_4_, 56.1 mL H_2_O, 70 °C. Silent conditions: Argon bubbling; US conditions: 547 kHz, P_acous_ = 55.5 W L^−1^ h^−1^.

Under low-frequency US (20 kHz), *tert-*butanol had a significant effect on benzyl alcohol conversion and benzaldehyde production, where conversion rates dropped from 43% to 5% and yield dropped from 34% to 5% (Figure 8). A similar drop in conversion rates and yield was observed for the transformation in silent conditions (from 37% to 5% for conversion, and from 29% to 5% for yield), suggesting that radical species are involved in the conversion of benzyl alcohol into benzaldehyde.

Under high-frequency US irradiation (547 kHz), *tert*-butanol also prevented transformations, with experimental results showing a decrease in conversion and benzaldehyde yield, respectively, from 39% to 5.0% and from 29% to 5.0%, as shown in Figure 8. The radical inhibitor prevented the transformation to occur, which indicates that radical species are involved in the mechanism. Since *tert*-butanol prevented transformations in silent conditions (drop in conversion and yield from 39% and 29% to 5% and 5%, respectively), this result shows that the radical species, which allow the transformation of benzyl alcohol to benzaldehyde, do not originate from low- or high-frequency US irradiation but rather from the Fenton system composed of FeSO_4_ and H_2_O_2_. The maximum theoretical hydroxyl production from the Fenton reagent reveals that 1.2 M of HO^•^ could be produced during the transformation as the reaction medium contains 584 mM of H_2_O_2_. This is approximately 20,000 times the concentration of hydroxyl radicals produced from a 547 kHz device in water for 15 min. This shows that *tert*-butanol inhibits benzyl alcohol oxidation to benzaldehyde in silent conditions as well as under US irradiation.

#### 2.8.2. Side Products and Transformation Pathways

**Figure 9:**
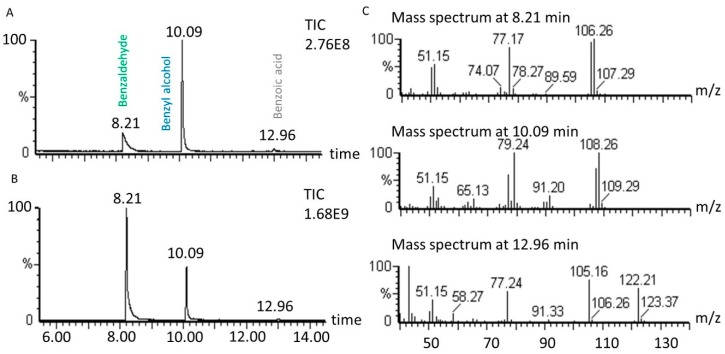
Oxidation of benzyl alcohol GC chromatogram (**A**) in silent conditions and (**B**) under US irradiation. (**C**) mass spectra of identified peaks obtained by electronic impact (70 eV). Reaction conditions under 20 kHz and silent conditions; 571 mM PhCH_2_OH, 1 molar equivalent of H_2_O_2_, 1% molar equivalent of catalyst, 5.61 mL H_2_O, 70 °C, 90 min. Silent conditions: Argon bubbling; US conditions: 20 kHz, P_acous_ = 47.9 W L^−1^ h^−1^, 13 mm probe.

Benzyl alcohol transformation leads to the production of benzaldehyde as the main product in the studied conditions. In some conditions under US, the selectivity for benzaldehyde is high (73%) with 41% benzyl alcohol conversion (1 molar equivalent of H_2_O_2_, 1% molar equivalent of catalyst, 5.61 mL H_2_O, 70 °C, 60 min). However, under silent conditions (1 molar equivalent of H_2_O_2_, 1% molar equivalent of catalyst, 5.61 mL H_2_O, 70 °C, 60 min) the transformation led to only 9% selectivity, with the reaction producing molecules other than benzaldehyde. Among organic products, benzoic acid represents less than 1% of benzyl alcohol conversion. To confirm benzaldehyde and benzoic acid production during the transformation, mass spectra obtained by GC-MS are presented in Figure 9.

No other significant organic compound was detected through LC-MS and GC-MS analyses, so the possibility for gas production was considered. Using γ-rays on ^14^C-labelled sodium benzoate, Matthews and Sangsters demonstrated that ^14^CO_2_ production, corresponding to benzoate decarboxylation, was initiated by hydroxyl radicals [51]. In another study, decarboxylation of benzoic acid at a concentration of 6.25 mM in aqueous medium at pH = 3 was obtained in the presence of hydrogen peroxide (1.68 molar equivalent relative to benzoic acid) and transition metal catalysts (FeSO_4_ and CuSO_4_; 4% molar equivalent) [52]. In our reaction conditions, benzoic acid can be decarboxylated leading to carbon dioxide production [53]. The release of CO_2_ to the atmosphere was studied by bubbling the reaction atmosphere in limewater. The lime water became cloudy, indicating that CO_2_ was produced during the transformation; no CO_2_ quantification was carried out. GC-MS analyses were conducted to look for benzene as a resulting product of decarboxylation, but no traces of this compound could be detected. Other peaks in the chromatograms with very low intensity could be detected but no further identification was applied in these cases.

#### 2.8.3. Comparison with Reported Mechanisms for the Oxidation of Benzyl Alcohol to Benzaldehyde

In literature, several mechanisms are described for the transformation of benzyl alcohol to benzaldehyde [17,19,32,40,43]. Narayanan et al. and Jia et al. propose three-step mechanisms involving ZMS-5 zeolite as a catalyst. [19,43]. They include a first activation step, the oxidation of the zeolite catalyst with H_2_O_2_ to peroxometal, which leads to a loss of a water molecule coming from the hydroperoxyl group and a proton of benzyl alcohol. A second loss of a proton from benzyl alcohol enables regeneration of the zeolite catalyst to its initial structure while benzaldehyde is generated. The major difference emerging from these two mechanisms is the origin of the proton implied in the formation of water and regeneration of the zeolite catalyst. Jia et al. proposed that the water molecule is formed notably from the alcohol moiety proton and the catalyst is regenerated from the methyl proton, whereas Narayanan et al. proposes the opposite. Others proposed a similar mechanism as Jia et al. but with tungstic acid as the catalyst [16].

Kamonsatikul et al. and Xiao et al. proposed similar mechanisms in which stabilizing ligands increase the adsorption of hydrogen peroxide on iron oxide catalysts to form hydroxyl radical species according to Fenton-like reactions [32,40]. The oxidation of benzyl alcohol would then occur through a simultaneous abstraction of protons from alcohol and methyl groups by hydroxyl radicals.

A system with conditions approaching ours, was described by Ahmad et al., involving 3 mmol of benzyl alcohol in 5 mL of water, 3 molar equivalents of H_2_O_2_, and 1%mol CuSO_4_ [17]. However, the reaction was not neutralized by a radical inhibitor, and UV-spectra measured during the reaction led the author to conclude that a single oxidation state Cu^II^ complex operated the transformation of benzyl alcohol to benzaldehyde through an ionic pathway.

Besides the implication of oxidizing radical species and catalyst stabilization, Mahamuni et al. considered the role of ultrasonic irradiation on benzyl alcohol transformation mechanisms to benzaldehyde [39]. They identified that gravitational collapse could enhance reactions because of the extreme temperature and pressure inside the bubble, but also by the increase of radicals in the bulk and the interface of the bubble. This interface is considered as a supercritical region where reactions can also take place. Ultrasonic irradiation of water also increases the concentration of hydroxyl radical species and increases the turbulence phenomenon that agitates the medium and raises the probabilities for radicals and benzyl alcohol to meet. In our case, the increased concentration of radical species was already discussed and it is not likely that the low concentration of radical species generated through US is the cause of the 45% increase in benzaldehyde yield. Negative synergism was described in sono-Fenton processes between chemical effects of US irradiation and Fenton processes because of HO^•^ scavenging from H_2_O_2_ [35]. The improvement for benzaldehyde selectivity under US is not clearly determined in our case but we could identify that low- and high-frequency US do not impact H_2_O_2_ decomposition and that at low frequency, US irradiation prevents benzaldehyde degradation.

## 3. Materials and Methods

### 3.1. Materials

Acetonitrile (HPLC grade) and manganese(IV) oxide (99%) were purchased from Fisher Scientific. Sodium sulfite, manganese(II) chloride, copper(II) chloride, and iron(III) chloride (all analysis grade) were purchased from Prolabo. Benzyl alcohol (99%), benzaldehyde (98%), iron powder 352 mesh (99%), sulfuric acid (96% in water), and potassium permanganate (98%) were procured from Acros. Iron(III) oxide (99.998%), iron(II, III) oxide (97%), iron(II) oxide (99.5%), manganese(II) oxide (99%), and manganese(II) titanium oxide (99.9%) were procured from Alfa Aesar. Copper(II) chloride (≥98%) and copper(II) sulfate (≥98%) came from Roth. Cobalt iron oxide (99%) was obtained from Aldrich. Benzoic acid (>99.5%), copper powder (grain size > 63 µm), and soda lime (both analysis grade) were obtained from Merck. Ultra-high-quality (UHQ) water obtained from Elga Maxima system was used for the experiments.

### 3.2. Equipement

Reactions under low-frequency US and controls were set up in 10 mL single neck round bottom flasks with a 20 kHz microtip device (Branson Digital Sonifier, nominal power of 200 W, 13 mm probe) operating at an acoustic power of 47.9 W L^−1^ h^−1^ according to calorimetric measurements with UHQ water. Reactions under high-frequency US were set up in cup horn reactors of 200-mL capacity. A NexTgen Ultrasonic Platform (SinapTec) device was used to generate US at 547 kHz. The acoustic power measured of water was 55.0 W L^−1^ h^−1^. Temperature was controlled during the transformation by a thermowell (SynapTec).

Carbon dioxide production was assessed using a two-neck, round bottom flask of 25-mL capacity related to argon bubbling and connected to a single-neck round bottom flask. Saturated limewater (23.3 mM) was used in the case of CO_2_ production assessment.

### 3.3. Experimental Procedures

For reactions under 20 kHz US irradiation, predetermined amounts of water, benzyl alcohol, and solid powder catalyst were introduced in the round-bottom flask. A predetermined volume of hydrogen peroxide 30% wt. was introduced as US irradiation started. Under silent conditions, argon bubbling was set up at an average flow rate of 6 L h^−1^. According to the desired hydrogen peroxide molar equivalent to substrate ratio, water volume was adapted to reach 6.366 mL as the final volume. Reactions under 547 kHz were directly set up in the cup-horn reactor. The introduction of reagents is similar to reaction under 20 kHz with a final volume of 63.66 mL.

At the end of each experiment, the whole volume of reaction medium is solubilized in acetonitrile (1:1 acetonitrile to reaction medium) and 200 µL of this mix is quenched with 200 mg of sodium sulfite.

Quenched samples were filtered with 0.22 µm PTFE filters (13 mm, Grosseron) and 1-mL luer syringes (Norm-Ject, Henke Sass Wolf) to remove solid particles.

H_2_O_2_ concentration monitoring in the medium was performed by indirect titration with KMnO_4_ at 2.5 × 10^−3^ M. For the experiment run under 20 kHz US irradiation and the associated silent control, the reaction medium was composed of 5.988 mL of water, 5.3 mg of FeSO_4_, and 378 µL of H_2_O_2_ 30% wt. The medium temperature was held at 70 °C and 300 µL were collected at 0, 5, 10, 15, 30, and 60 min. The sample was solubilized in 10 mL of water and 500 µL of H_2_SO_4_ (96% in water). The solution of KMnO4 at 2.5 × 10^−3^ M was added in the sample preparation, and the volume used to reduce MnO_4_^−^ to Mn^2+^ was identified to calculate H_2_O_2_ concentration. For the experiment run under 547 kHz and the silent associated control, the medium was composed of 59.88 mL of water, 53.0 mg of FeSO_4_, and 3.78 mL of H_2_O_2_ 30% wt. The same procedure as described above was performed. Hydrogen peroxide concentration was monitored over 120 min in this case.

### 3.4. Experimental Analysis

An HPLC system (Perkin Elmer Series 200) equipped with a diode array UV-visible detector (Perkin Elmer Series 200, λ = 258 nm) and a 3 μm Surf C18 TriF 100A (ImChem, 33 × 4.6 mm) column was used to monitor benzyl alcohol, benzaldehyde, and benzoic acid concentrations. Mobile phase was composed of water with 0.1% acetic acid and acetronitrile and started with 10% of acetonitrile (1 min), reaching 100% within 20 min (5 min). Pure samples of benzyl alcohol, benzaldehyde, and benzoic acid of known concentrations were prepared for calibration and quantification. For benzaldehyde, a non-linear response was observed in the studied range and calibration curves were defined for several ranges of concentrations with good reliability (R^2^ > 0.95). Benzyl alcohol and benzoic acid response to concentration was linear (R^2^ > 0.95) in the studied range.

GC-MS analyses were conducted on a Clarus 600 gas chromatograph and a Clarus 600 T mass spectrometer (Perkin Elmer) using an Optima 5 MS accent column (30 m × 0.25 mm i.d., 0.25 µm film thickness, Macherey–Nagel). The column oven temperature started at 50 °C and was held for 5 min. The temperature was increased at a rate of 8 °C/min until it reached 180 °C and was then held for 5 min. Then, it was increased at a rate of 10 °C/min until it reached 340 °C and was held for 1 min. The total run time was 42.25 min. Injection and transfer line temperatures were set up at 200 °C and 300 °C, respectively. Mass spectrometry was obtained by electronic impact ionization in positive mode detection, with the ion source at 250 °C and the detector voltage set at 70 eV.

## 4. Conclusions

In this study, we investigated simple and environmentally sustainable reaction conditions to selective oxidize benzyl alcohol into benzaldehyde. Various parameters such as substrate concentration, oxidant nature and amount, catalyst nature and amount, temperature, pH, reaction duration, and US frequency were studied over wide ranges to identify optimal conditions for the transformation. We identified that a concentration of 571 mM of benzyl alcohol in acidic water at 70 °C for 15 min in the presence of 1% molar equivalent of FeSO_4_ as catalyst and 1 molar equivalent of H_2_O_2_ as oxidizing agent, enabled 39% conversion of benzyl alcohol and 20% yield of benzaldehyde, which corresponds to 51% selectivity for benzaldehyde. The irradiation under 20 kHz US led to a significant increase in selectivity of 73% with a similar conversion rate of 39%. Study of the mechanism indicated that radical species such as HO^•^, produced by Fenton reagents, are implied in the oxidation of benzyl alcohol to benzaldehyde. Low-frequency US irradiation led to stable concentrations of benzaldehyde over time, presumably by preventing benzaldehyde degradation in the reaction medium. After 60 min under 547 kHz US irradiation, benzyl alcohol conversion was increased by +3% and benzaldehyde yield by +23%, confirming a beneficial effect of US irradiation of either low or high frequency on benzaldehyde selectivity (+43% after 15 min and +19% after 120 min, respectively) in the oxidation of benzyl alcohol.

This simple system implemented green oxidizing agent and catalyst and was developed to improve selective oxidation of benzyl alcohol as a well-known substrate in the model transformation to benzaldehyde. An exciting method to improve the system in an environmentally responsible manner would be to combine US irradiation with UV-light irradiation. Synergistic effects have already been described in several fields such as organic pollutant mineralization [54,55], biomass treatment [56], and green organic chemistry [57]. Beneficial effects on selective oxidation could emerge from the association of US and UV as green techniques and have yet to be studied.

## Figures and Tables

**Table 1:** Effect of benzyl alcohol concentration on its conversion rate and benzaldehyde yield. Reaction conditions: 1 molar eq. H_2_O_2_; 1% molar eq. FeSO_4_; 70 °C; 15 min. Silent conditions: Argon bubbling; ultrasound (US) conditions: 20 kHz, P_acous_ = 47.9 W L^−1^ h^−1^, 13 mm probe.

In Silent Conditions				Under US Irradiation		
[PhCH_2_OH] (mM)	PhCH_2_OH Conversion (%)	PhCHO Yield (%)	PhCHO Selectivity (%)	PhCH_2_OH Conversion (%)	PhCHO Yield (%)	PhCHO Selectivity (%)
286	43.8	12.2	28.0	51.1	19.0	37.1
571	54.8	12.7	23.2	59.3	21.2	35.7
1142	46.7	7.7	16.5	49.3	12.9	26.2
2284	49.2	6.1	12.5	47.9	7.1	14.9

**Table 2:** Oxidation of PhCH_2_OH according to nature of catalyst: Mineral metal oxides and ionic crystals in silent condition and under US irradiation (20 kHz). Reaction conditions: 571 mM PhCH_2_OH, 1 molar eq. H_2_O_2_; 1% molar eq. catalyst, 5.61 mL H_2_O, 70 °C, 15 min. Silent conditions: Argon bubbling; US conditions: 20 kHz, P_acous_ = 47.9 W L^−1^ h^−1^, 13 mm probe.

	In Silent Conditions				Under US Irradiation		
	Catalyst	PhCH_2_OH Conversion (%)	PhCHO Yield (%)	PhCHO Selectivity (%)	PhCH_2_OH Conversion (%)	PhCHO Yield (%)	PhCHO Selectivity (%)
Metal oxides	Fe_3_O_4_	18.2	0.3	1.5	17.0	0.6	3.4
	FeO	19.8	0.2	0.9	17.6	0.2	1.1
	Fe_2_O_3_	17.1	0.1	0.8	15.6	0.2	1.1
	FeTiO_3_	18.0	0.3	1.5	16.4	0.5	3.1
	CoFeO_4_	18.4	0.1	0.6	16.7	0.3	1.6
	MnTiO_3_	19.5	0.2	0.9	18.1	0.2	1.1
Metals	Cu	25.8	8.0	30.9	17.1	4.2	24.7
	Fe	13.4	0.5	3.4	11.5	2.1	18.2
Salts	MnCl_2_	11.8	0.4	3.1	15.0	2.5	16.4
	CuCl_2_	16.4	4.6	27.8	16.2	6.8	41.7
	CuSO_4_	10.8	4.6	42.4	10.6	8.1	77.0
	FeCl_3_	50.2	3.4	6.8	52.6	16.9	32.2
	FeSO_4_	40.4	19.6	48.5	43.3	26.2	60.5

**Table 3:** Oxidation of PhCH_2_OH according to nature of the catalyst and H_2_O_2_ concentration in silent condition and under US irradiation (20 kHz). Reaction conditions: 571 mM PhCH_2_OH 1% molar eq. catalyst, 5.610 mL H_2_O, 70 °C, 15 min. Silent conditions: Argon bubbling; US conditions: 20 kHz, P_acous_ = 47.9 W L^−1^ h^−1^, 13 mm probe.

	eq. Ox	PhCH_2_OH Conversion	PhCHO Yield	PhCHO Seletivity	eq. Ox	PhCH_2_OH Conversion	PhCHO Yield	PhCHO Seletivity
		FeSO_4_				CuSO_4_		
Silent	0.5	26.2%	12.1%	46.1%	0.5	14.5%	2.9%	20.3%
	1	40.4%	19.6%	48.5%	1	10.8%	4.6%	42.4%
	2	70.9%	9.3%	13.2%	2	20.9%	5.4%	26.0%
US (20 kHz)	0.5	28.4%	13.8%	48.5%	0.5	15.2%	5.3%	34.8%
	1	43.3%	26.2%	60.5%	1	10.6%	8.1%	77.0%
	2	75.4%	15.8%	21.0%	2	18.9%	7.9%	42.0%
		FeCl_3_				CuCl_2_		
Silent	0.5	25.5%	6.9%	26.9%	0.5	15.2%	3.4%	22.4%
	1	50.2%	3.4%	6.8%	1	16.4%	4.6%	27.8%
	2	74.5%	2.0%	2.6%	2	20.5%	5.3%	25.8%
US (20 kHz)	0.5	25.0%	15.0%	60.2%	0.5	16.5%	5.2%	31.5%
	1	52.6%	16.9%	32.2%	1	16.2%	6.8%	41.7%
	2	66.1%	16.8%	25.4%	2	19.2%	7.8%	40.7%
		Fe				Cu		
Silent	1	11.8%	0.4%	3.1%	0.5	8.8%	1.1%	13.0%
	2	8.7%	0.4%	4.2%	1	25.8%	8.0%	30.9%
	3	7.8%	0.4%	4.8%	2	15.0%	5.6%	37.3%
US (20 kHz)	1	11.5%	2.1%	18.2%	0.5	22.6%	5.6%	24.5%
	2	8.4%	1.0%	11.8%	1	17.1%	4.2%	24.7%
	3	11.2%	0.7%	6.2%	2	16.5%	10.1%	61.5%
		MnCl_2_						
Silent	0.5	6.1%	0.3%	4.9%				
	1	6.4%	0.5%	7.2%				
	2	8.8%	1.1%	13.0%				
US (20 kHz)	0.5	9.8%	1.8%	17.9%				
	1	7.9%	2.5%	30.9%				
	2	6%	1%	14%				

## References

[1] Lenoir D. (2006). Selective Oxidation of Organic Compounds—Sustainable Catalytic Reactions with Oxygen and without Transition Metals?. Angew. Chem. Int. Ed..

[2] Dugger R.W., Ragan J.A., Ripin D.H.B. (2005). Survey of GMP Bulk Reactions Run in a Research Facility between 1985 and 2002. Org. Process Res. Dev..

[3] Carey J.S., Laffan D., Thomson C., Williams M.T. (2006). Analysis of the Reactions Used for the Preparation of Drug Candidate Molecules. Org. Biomol. Chem..

[4] Penteado F., Monti B., Sancineto L., Perin G., Jacob R.G., Santi C., Lenardão E.J. (2018). Ultrasound-Assisted Multicomponent Reactions, Organometallic and Organochalcogen Chemistry. Asian J. Org. Chem..

[5] Chatel G. (2017). How Sonochemistry Contributes to Green Chemistry?. Ultrason. Sonochem..

[6] Cravotto G., Cintas P. (2007). Forcing and Controlling Chemical Reactions with Ultrasound. Angew. Chem. Int. Ed..

[7] Luche J.L. (1998). Synthetic Organic Sonochemistry.

[8] Colarusso P., Serpone N. (1996). Sonochemistry II.—Effects of Ultrasounds on Homogeneous Chemical Reactions and in Environmental Detoxification. Res. Chem. Intermed..

[9] Amaniampong P.N., Karam A., Trinh Q.T., Xu K., Hirao H., Jérôme F., Chatel G. (2017). Selective and Catalyst-Free Oxidation of D-Glucose to D-Glucuronic Acid Induced by High-Frequency Ultrasound. Sci. Rep..

[10] Suslick K.S., Meyers R.A. (2001). Sonoluminescence and Sonochemistry. Encyclopedia of Physical Science and Technology.

[11] Mason T.J. (2003). Sonochemistry and Sonoprocessing: The Link, the Trends and (Probably) the Future. Ultrason. Sonochem..

[12] Fang X., Mark G., von Sonntag C. (1996). OH Radical Formation by Ultrasound in Aqueous Solutions Part I: The Chemistry Underlying the Terephthalate Dosimeter. Ultrason. Sonochem..

[13] Ross A.B., Farhataziz A. (1977). Selective specific rates of reactions of transients in water and aqueous solutions. Part III. Hydroxyl Radical and Perhydroxyl Radical and Their Radical Ions.

[14] Anastas P., Warner J. (2000). Green Chemistry: Theory and Practice.

[15] Matsumoto T., Ueno M., Wang N., Kobayashi S. (2008). Recent Advances in Immobilized Metal Catalysts for Environmentally Benign Oxidation of Alcohols. Chem. Asian J..

[16] Chaudhari M.P., Sawant S.B. (2004). Kinetics of Heterogeneous Oxidation of Benzyl Alcohol with Hydrogen Peroxide. Chem. Eng. J..

[17] Ahmad J.U., Räisänen M.T., Leskelä M., Repo T. (2012). Copper Catalyzed Oxidation of Benzylic Alcohols in Water with H_2_O_2_. Appl. Catal. A.

[18] Zhan G., Huang J., Du M., Sun D., Abdul-Rauf I., Lin W., Hong Y., Li Q. (2012). Liquid Phase Oxidation of Benzyl Alcohol to Benzaldehyde with Novel Uncalcined Bioreduction Au Catalysts: High Activity and Durability. Chem. Eng. J..

[19] Jia A., Lou L.L., Zhang C., Zhang Y., Liu S. (2009). Selective Oxidation of Benzyl Alcohol to Benzaldehyde with Hydrogen Peroxide over Alkali-Treated ZSM-5 Zeolite Catalysts. J. Mol. Catal. A Chem..

[20] Mason J.T., Cintas P. (2002). Sonochemistry. Handbook of Green Chemistry and Technology.

[21] Mason T.J. (2007). Sonochemistry and the Environment–Providing a “Green” Link between Chemistry, Physics and Engineering. Ultrason. Sonochem..

[22] Noyori R., Aoki M., Sato K. (2003). Green Oxidation with Aqueous Hydrogen Peroxide. Chem. Commun..

[23] ten Brink G.J., Arends I.W.C.E., Sheldon R.A. (2004). The Baeyer−Villiger Reaction:  New Developments toward Greener Procedures. Chem. Rev..

[24] Zhan B.-Z., Thompson A. (2004). Recent Developments in the Aerobic Oxidation of Alcohols. Tetrahedron.

[25] Brzaszcz M., Kloc K., Maposah M., Mlochowski J. (2000). Selenium(IV) Oxide Catalyzed Oxidation of Aldehydes to Carboxylic Acids with Hydrogen Peroxide. Synth. Commun..

[26] Sancineto L., Tidei C., Bagnoli L., Marini F., Lenardão E.J., Santi C. (2015). Selenium Catalyzed Oxidation of Aldehydes: Green Synthesis of Carboxylic Acids and Esters. Molecules.

[27] Shul’pin G.B., Bryliakov K.P. (2019). Metal.-catalyzed oxidation of C–H compounds with peroxides in unconventional solvents. Frontiers of Green Catalytic Selective Oxidations.

[28] Levitsky M.M., Bilyachenko A.N., Shul’pin G.B. (2017). Oxidation of C-H Compounds with Peroxides Catalyzed by Polynuclear Transition Metal Complexes in Si- or Ge-Sesquioxane Frameworks: A review. J. Organomet. Chem..

[29] Shul’pin G.B. (2016). New Trends in Oxidative Functionalization of Carbon–Hydrogen Bonds: A Review. Catalysts.

[30] Mahmudov K.T., Gurbanov A.V., Guseinov F.I., Guedes da Silva M.F.C. (2019). Noncovalent Interactions in Metal Complex Catalysis. Coord. Chem. Rev..

[31] Nesterov D.S., Nesterova O.V., Pombeiro A.J.L. (2018). Homo- and Heterometallic Polynuclear Transition Metal Catalysts for Alkane CH Bonds Oxidative Functionalization: Recent Advances. Coord. Chem. Rev..

[32] Shi F., Tse M.K., Pohl M.M., Brückner A., Zhang S., Beller M. (2007). Tuning Catalytic Activity between Homogeneous and Heterogeneous Catalysis: Improved Activity and Selectivity of Free Nano-Fe_2_O_3_ in Selective Oxidations. Angew. Chem..

[33] Kamonsatikul C., Khamnaen T., Phiriyawirut P., Charoenchaidet S., Somsook E. (2012). Synergistic Activities of Magnetic Iron-Oxide Nanoparticles and Stabilizing Ligands Containing Ferrocene Moieties in Selective Oxidation of Benzyl Alcohol. Catal. Commun..

[34] Andreozzi R., Caprio V., Insola A., Marotta R. (1999). Advanced Oxidation Processes (AOP) for Water Purification and Recovery. Catal. Today.

[35] Chakma S., Moholkar V.S. (2013). Physical Mechanism of Sono-Fenton Process. AIChE.

[36] Pourbaix M. (1974). Atlas of Electrochemical Equilibria in Aqueous Solutions.

[37] Wang S. (2008). A Comparative Study of Fenton and Fenton-Like Reaction Kinetics in Decolourisation of Wastewater. Dyes Pigm..

[38] Jones C.W. (1999). Applications of Hydrogen Peroxide and Its Derivatives; Clean Technology Monographs.

[39] Mahamuni N.N., Gogate P.R., Pandit A.B. (2006). Ultrasonic Synthesis of Benzaldehyde from Benzyl Alcohol Using H_2_O_2_: Role of Ultrasound. Ind. Eng. Chem. Res..

[40] Xiao S., Zhang C., Chena R., Chen F. (2015). Selective Oxidation of Benzyl Alcohol to Benzaldehyde with H_2_O_2_ in Water on Epichlorohydrin-Modified Fe_3_O_4_ Microspheres. New J. Chem..

[41] Wang X., Wu G., Li J., Zhao N., Wei W., Sun Y. (2007). Surface-Modified Improvement in Catalytic Performance of Cr(Salen) Complexes Immobilized on MCM-41 in Solvent-Free Selective Oxidation of Benzyl Alcohol. Catal. Lett..

[42] Long N.Q., Quan N.A. (2014). Highly Selective Oxidation of Benzyl Alcohol to Benzaldehyde Catalyzed by Nano Au/Γ-Al_2_O_3_ under Environment-Friendly Conditions. React. Kinet. Catal. Lett..

[43] Narayanan S., Vijaya J.J., Sivasanker S., Kennedy L.J., Ariharan A. (2014). Enhanced Selectivity to Benzaldehyde in the Liquid Phase Oxidation of Benzyl Alcohol Using Nanocrystalline ZSM-5 Zeolite Catalyst. J. Porous Mater..

[44] Yalkowsky S.H., He Y., Jain P. (2019). Handbook of Aqueous Solubility Data.

[45] Kuo W.G. (1992). Decolorizing Dye Wastewater with Fenton’s Reagent. Water Res..

[46] Yoon J., Lee Y., Kim S. (2001). Investigation of the Reaction Pathway of OH Radicals Produced by Fenton Oxidation in the Conditions of Wastewater Treatment. Water Sci. Technol..

[47] Zhou T., Li Y., Ji J., Wong F.-S., Lu X. (2008). Oxidation of 4-Chlorophenol in a Heterogeneous Zero Valent Iron/H_2_O_2_ Fenton-Like System: Kinetic, Pathway and Effect Factors. Sep. Purif. Methods.

[48] Entezari M.H., Kruus P. (1994). Effect of Frequency on Sonochemical Reactions. I: Oxidation of Iodide. Ultrason. Sonochem..

[49] Tauber A., Mark G., Schuchmann H.-P., Von Sonntag C. (1999). Sonolysis of *Tert*-Butyl Alcohol in Aqueous Solution. J. Chem. Soc. Perkin Trans..

[50] Hamdaoui O., Naffrechoux E. (2008). Sonochemical and Photosonochemical Degradation of 4-Chlorophenol in Aqueous Media. Ultrason. Sonochem..

[51] Matthews R.W., Sangster D.F. (1965). Measurement by Benzoate Radiolytic Decarboxylation of Relative Rate Constants for Hydroxyl Radical Reactions. J. Phys. Chem..

[52] Gardner L.K., Lawrence G.D. (1993). Benzene Production from Decarboxylation of Benzoic Acid in the Presence of Ascorbic Acid and a Transition-Metal Catalyst. J. Agric. Food Chem..

[53] Lamrinia R., Lacan P., Francina A., Guilluy R., Desage M., Michon J., Becchid M., Brazierb J.L. (1998). Oxidative Decarboxylation of Benzoic Acid by Peroxyl Radicals. Free Radical Biol. Med..

[54] Naffrechoux E., Chanoux S., Petrier C., Suptil J. (2000). Sonochemical and Photochemical Oxidation of Organic Matter. Ultrason. Sonochem..

[55] Joseph C.G., Li Puma G., Bono A., Krishnaiah D. (2009). Sonophotocatalysis in Advanced Oxidation Process: A Short Review. Ultrason. Sonochem..

[56] Chatel G., Valange S., Behling R., Colmenares J.C. (2017). A Combined Approach Using Sonochemistry and Photocatalysis: How to Apply Sonophotocatalysis for Biomass Conversion?. ChemCatChem.

[57] Naruke Y., Tanaka H., Harada H. (2011). The Effects of Coupling Photocatalysis and Sonolysis on Malonic Acid Solution. Electrochemistry.

